# Ambient Air Pollution and the Progression of Atherosclerosis in Adults

**DOI:** 10.1371/journal.pone.0009096

**Published:** 2010-02-08

**Authors:** Nino Künzli, Michael Jerrett, Raquel Garcia-Esteban, Xavier Basagaña, Bernardo Beckermann, Frank Gilliland, Merce Medina, John Peters, Howard N. Hodis, Wendy J. Mack

**Affiliations:** 1 Swiss Tropical and Public Health Institute (Swiss TPH), Basel, Switzerland; 2 Centre for Research in Environmental Epidemiology CREAL, Barcelona, Spain; 3 Division of Environmental Health Sciences, School of Public Health, University of California, Berkeley, California, United States of America; 4 Department of Preventive Medicine, University of Southern California, Los Angeles, California, United States of America; 5 Atherosclerosis Research Unit, Department of Medicine, University of Southern California, Los Angeles, California, United States of America; University of Cape Town, South Africa

## Abstract

**Background:**

Cross-sectional studies suggest an association between exposure to ambient air pollution and atherosclerosis. We investigated the association between outdoor air quality and progression of subclinical atherosclerosis (common carotid artery intima-media thickness, CIMT).

**Methodology/Principal Findings:**

We examined data from five double-blind randomized trials that assessed effects of various treatments on the change in CIMT. The trials were conducted in the Los Angeles area. Spatial models and land-use data were used to estimate the home outdoor mean concentration of particulate matter up to 2.5 micrometer in diameter (PM2.5), and to classify residence by proximity to traffic-related pollution (within 100 m of highways). PM2.5 and traffic proximity were positively associated with CIMT progression. Adjusted coefficients were larger than crude associations, not sensitive to modelling specifications, and statistically significant for highway proximity while of borderline significance for PM2.5 (P = 0.08). Annual CIMT progression among those living within 100 m of a highway was accelerated (5.5 micrometers/yr [95%CI: 0.13–10.79; p = 0.04]) or more than twice the population mean progression. For PM2.5, coefficients were positive as well, reaching statistical significance in the socially disadvantaged; in subjects reporting lipid lowering treatment at baseline; among participants receiving on-trial treatments; and among the pool of four out of the five trials.

**Conclusion:**

Consistent with cross-sectional findings and animal studies, this is the first study to report an association between exposure to air pollution and the progression of atherosclerosis – indicated with CIMT change – in humans. Ostensibly, our results suggest that air pollution may contribute to the acceleration of cardiovascular disease development – the main causes of morbidity and mortality in many countries. However, the heterogeneity of the volunteering populations across the five trials, the limited sample size within trials and other relevant subgroups, and the fact that some key findings reached statistical significance in subgroups rather than the sample precludes generalizations to the general population.

## Introduction

Cardiovascular disease (CVD) is the most important cause of morbidity and mortality in the developed world, and atherosclerosis is the central underlying pathology [1]. Atherogenesis is a life-long process involving a range of mechanisms including lipid peroxidation and inflammation affecting the vascular wall. The clinically most relevant results of this pathology are myocardial infarction and stroke. Evidence for acute cardiovascular effects of air pollution has substantially increased in recent years; most experts agree that ambient particulate matter plays a role in triggering cardiovascular events among those with predisposing cardiovascular pathologies [2], [3]. Experimental studies indicate a mechanistic role of air pollution in various acute cardiovascular processes including ischemia, endothelial dysfunction, activation of the fibrinolytic system and possibly plaque destabilization[2], [4].

Far less research has tested the question whether ambient air pollution also contributes to the development of chronic pathologies, such as atherosclerosis, that predispose to acute cardiovascular disease. Animal studies conducted in rabbits, rats or mice with urban particulate matter instillation and inhalation experiments, indicate more rapid progression of atherosclerosis among animals exposed to urban particulate matter (PM) compared to filtered air [4], [5], [6], [7]. A few cross-sectional studies observed significant associations between ambient PM or other markers of air pollution and the degree of atherosclerosis, measured with carotid artery intima-media thickness (CIMT) [8], and both coronary and aortic calcifications [9], [10]. To our knowledge, no study has investigated the impact of ambient air pollution on the progression of atherosclerosis in humans. Herein we used longitudinal data available from five randomized controlled trials to investigate whether chronic exposures to ambient air pollutants contribute to a faster progression rate of atherosclerosis in humans. We have previously reported a cross-sectional analysis of baseline CIMT from two of the five trials in the current study and observed a significant cross-sectional association between CIMT levels at baseline and assigned home outdoor levels of ambient PM2.5 [8]. We added longitudinal data from three additional trials that have completed two to three years of average follow-up [11], [12], [13], [14].

## Methods

### Trials

This study used data from 5 pre-existing clinical trials, all of which are registered in ClinicalTrials.gov. (a) the B-Vitamin Atherosclerosis Intervention Trial (BVAIT; NCT 00114400 [http://clinicaltrials.gov/ct2/show/NCT00114400])[14]; (b) the Vitamin E Atherosclerosis Prevention Study (VEAPS; NCT 00114387 [http://clinicaltrials.gov/ct2/show/NCT00114387]) [15]; (c) the Estrogen in the Prevention of Atherosclerosis Trial (EPAT; NCT 00115024 [http://clinicaltrials.gov/ct2/show/NCT00115024]) [12]; (d) the Troglitazone Atherosclerosis Regression Trial (TART [NCT 00116545; http://clinicaltrials.gov/ct2/show/NCT00116545]) [11]; and (e) the Women's Estrogen-Progestin Lipid-Lowering Hormone Atherosclerosis Regression Trial (WELL-HART; NCT 00000559 [http://clinicaltrials.gov/ct2/show/NCT00000559]) [13]. Table 1 summarizes the main characteristics of these trials and more details are given elsewhere [11], [12], [13], [14], [15]. (For exclusion criteria of each trial, see supplemental material, Table S2). All trials were conducted with adult volunteers. Inclusion criteria differed for each trial according to the primary hypothesis and the intervention of interest, namely testing the anti-atherogenic property of hormone replacement therapy [12], [13], vitamin supplementation [14], [15], and a diabetes treatment [11]. All clinical trials used in these analyses were approved by the institutional review board of the University of Southern California (USC).

**Table 1:** Description of the B-Vitamin Atherosclerosis Intervention Trial (BVAIT), the Vitamin E Atherosclerosis Prevention Study (VEAPS), the Estrogen in the Prevention of Atherosclerosis Trial (EPAT), the Troglitazone Atherosclerosis Regression Trial (TART), and the Women's Estrogen-Progestin Lipid-Lowering Hormone Atherosclerosis Regression Trial (WELL-HART) used in this analyses.

Characteristic	BVAIT	VEAPS	EPAT	TART	WELLHART
Year (start / end of trial)	2001 / 2006	1996 / 2000	1994 / 1998	1997 / 2000	1995 / 2000
Mean (range) follow-up duration in yrs	3.3 (0.5–5.1)	2.8 (0.5–3.5)	1.8 (0.4–2.4)	1.8 (0.4–2.4)	2.9 (0.4–4.0)
N at baseline	506	353	222	299	226
N with progression & outdoor PM2.5 data	475	350	169	275	214
Mean age (range) at baseline	61.4 (40–89)	56.2 (40–82)	61.4 (47–80)	52.6 (30–71)	63.7 (49–76)
On-trial Intervention (tx)	Vitamin B	Vitamin E (α-Tocopherol)	Unopposed estrogen	Troglitazone (thiazolidinedione)	Estrogen/Estrogen-Progestin
Main inclusion Criteria					
Sex	Men & post-menopausal Women	Men & Women	Postmenopausal women	Men & Women	Postmenopausal Women
Age	>40 yrs	>40 yrs	≥45 yrs	30–70 yrs	≤75 yrs
Other	fasting plasma homocysteine ≥1.15 md/dL	LDL >130 mg/dL	LDL ≥130 mg/dL	diagnosed w/ type 2 diabetes mellitus onset at ≥30 yrs of age; fasting glucose >140 mg/dL & <350 mg/dL; receiving <150 units insulin/day.	LDL = 100–250 mg/dL; ≥1 coronary artery lesion
Main reference	Hodis et al, 2009 [14]	Hodis et al, 2002, [15]	Hodis et al, 2001, [12]	Hodis et al, 2006, [11]	Hodis et al, 2003, [13]

(CVD  =  cardiovascular diseases; LDL  =  low density lipids). For exclusion criteria: see Table S2 in the online supplement.

For our study, the main outcome of interest is the change over time in the carotid artery intima-media thickness (CIMT). Repeated measurements of CIMT are a well-established non-invasive method to test the effect of interventions on atherosclerosis. In all trials, high-resolution far-wall B-mode ultrasound images of the right common carotid artery (CCA) to measure far wall CIMT were obtained at baseline prior to randomization to treatment, and every 6 months thereafter for the duration of the trials, using the same ultrasound hardware and software and ultrasonographers during the full follow-up. Details of this highly-reproducible method are published [15], [16], [17]. Blood pressure, height and weight were measured using standard procedures. The baseline questionnaires included an assessment of all major cardiovascular disease risk factors and potential confounding variables. The current residential address was available for each subject. Fasting blood samples were drawn for lipid measurements. Data used in our analyses were collected uniformly with the same instruments in each trial. We used two main markers of exposure to ambient air pollution to assign long-term residential exposure to each subject, namely the modeled average home outdoor level of PM2.5 and the distance of the residence from traffic corridors, a widely used marker of exposure to unmeasured traffic-related pollutant concentrations [18].

### Exposure to ambient PM2.5

Estimates of the home outdoor mean PM2.5 concentrations were assigned as described in our previous cross-sectional analysis [8], [19]. In brief, a PM2.5 surface was derived from a geo-statistical model with data derived from 23 state and local district monitoring stations (year 2000). To assign exposure, PM_2.5_ data were interpolated using a combination of a universal kriging model with a quadratic drift and a multiquadric radial basis function model [20], [21]. These models do not take into account local information such as traffic density or other metrics. We averaged the two surfaces based on 25-meter grid cells. We linked the address of each subject with the exposure surface through a geo-coding database (www.esri.com). All models were implemented with ArcScript from the Environmental Systems Research Institute (ESRI, Redlands, CA).

### Distance to highway or main road

In line with the known spatial distributions of traffic-related pollutants and previously published studies, we defined the 100-meter corridor along highways as a ‘high exposure zone’. Those 23 subjects (1.5% of all) living further than the 100-meter corridor are assumed to have ‘background’ level exposure to these same pollutants [18]. To increase the number of subjects falling into the ‘high exposure zone’ and provide comparability with other studies examining cardiovascular endpoints [22], [23], we also used *proximity to highways (100 m) or living within 50 m of a major road* as a marker of exposure. Traffic density, and in particular diesel truck density, is much lower on main roads than on highways; thus, these 238 ‘exposed’ subjects (16.1%) experienced, on average, lower concentrations of traffic-related pollutants than the subset living within 100 m of highways.

### Statistical models

To make use of the complete follow-up data, missing covariate data were imputed employing standard methods [24]. The missing data structure and the particular method of imputation we used are described in the supplement (Text S1 and Table S1). As a result, all models used the same complete data set of N = 1483. The main model presented in the results included only 5 subjects with one or more imputed covariates. Thus, results among those with complete data and the total sample including the five were identical.

Our main outcome was the annual rate of change in CIMT, derived for each subject as the slope coefficient from the linear regression across all CIMT measurements against follow-up time (in years). Using this outcome variable, we fitted separate linear regression models for PM2.5 and road proximity metrics, adjusted for baseline covariates. Beta coefficients are expressed as the annual change in CIMT (micrometers/year) per a 10 ug/m^3^ contrast in PM2.5 and for living in proximity to a highway or main road (yes/no). While treatments differed across the trials, all studies investigated the potential effects of treatment on CIMT progression. Thus, all models included one indicator variable to control for treatment. We explored the sensitivity of the results to adjustment for several sets of covariates, including trial. Given that the causal paths linking pollution with atherogenesis may be complex, we also evaluated the impact of adjustment for co-variates that may be markers of factors on the causal paths, e.g. hypertension, physical activity, overweight, menopause and others. The impact of adjustment for CIMT at baseline was also tested, and *trial* was alternatively included as a fixed and random effect, respectively. While we present results for a range of models, we chose a parsimonious model as the ‘main model’, with adjustment for treatment status, smoking, wine consumption, height, education, and reported lipid lowering treatments.

Since our data came from five different trials with different inclusion criteria, we also analyzed each trial separately and then combined the results using a random-effects meta-analysis [25]. Results presented in the figures utilized this approach. We tested heterogeneity of the estimates using the Cochran's chi-square test or *Q*-test.

Our previously published cross-sectional findings from two trials gave some indication of possibly different associations between air pollution and baseline CIMT among subgroups defined by sex, age, and history of lipid-lowering therapy (taken as a marker for being at greater risk for atherosclerosis). In our longitudinal analyses, interactions of the main effects of each of the exposures with these factors were tested. We also tested whether the main effects differed by on-trial treatment and by markers of socio-economic status (income and education level). Several U.S. air pollution studies observed larger effects of pollution among socially disadvantaged groups [19].

We also present “two-pollutant models” with both PM2.5 and highway proximity included in the model, assuming that the two markers may capture different (interrelated) aspects of ambient air pollution, namely urban background pollution (mostly due to secondary pollutants) and the near-source traffic-related pollutants with higher concentrations of primary pollutants.

## Results

Table 2 presents the main analytic variables for the populations of each trial. As expected, the heterogeneous inclusion criteria across the five trials result in several differences between trials. As usual in longitudinal CIMT studies of only a few years, CIMT progressed, on average, but decreased in many subjects. The mean annual progression of CIMT was 2.04 µm/year (standard deviation of ±12.91 µm/year). While home outdoor PM2.5 concentrations were comparable across trials, none of the EPAT subjects lived within 100 m of a highway.

**Table 2:** Descriptive summary of the study population including only subjects with both CIMT progression and home outdoor PM2.5 data (N = 1483).

Characteristic	BVAIT	VEAPS	EPAT		TART	WELLHART	TOTAL SAMPLE	
	(N = 475)	(N = 350)	(N = 169)		(N = 275)	(N = 214)	(N = 1483)	
CIMT at baseline in mm (mean ±sd; range)	0.75±0.14	0.76±0.13	0.76±0.13		0.82±0.15	0.84±0.20	0.78±0.15	
	(0.50–1.51)	(0.55–1.26)	(0.51–1.55)		(0.53–1.36)	(0.55–2.09)	(0.50–2.09)	
CIMT progression / yr in mcrm (mean ±sd; range)	2.50±7.47	2.85±8.46	0.68±15.55		4.71±21.22	−2.68±10.63	2.04±12.91	
	(−24.90, 28.43)	(−31.54, 29.86)	(−35.15, 49.56)		(−34.27, 40.65)	(−44.08, 43.09)	(−44.08, 49.56)	
PM_2.5_ mean ±sd; (25th–75th percentile)	20.12±2.82	20.66±2.19	21.20±2.09		21.87±1.10	20.80±2.44	20.79±2.37	
	(19.22–21.94)	(20.28–21.84)	(20.92–22.08)		(21.51–22.53)	(20.69–22.11)	(20.49–22.05)	
Living within 100 m of highway, N (%)	9 (1.89)	5 (1.43)	0 (0.00)		7 (2.55)	2 (0.93)	23 (1.55)	
Living within 100 m of highway or within 50 m of a main road, N (%)	70 (14.74)	50 (14.29)	23 (13.61)		59 (21.45)	36 (16.82)	238 (16.05)	
Age; mean ±sd; (range)	61.38±9.93	56.19±8.94	61.35±6.82		52.56±8.96	63.66±6.47	58.85±9.57	
	(40.21–88.59)	(39.55–82.03)	(48.00–80.00)		(29.72–70.95)	(49.00–76.00)	(29.72–88.59)	
% Women	39.37	52.00	100.00		67.27	100.00	63.18	
% Women in menopause	100.00	66.11	100.00		73.51	100.00	68.17	
Highest Education (%)								
HS graduate or less	6.54	7.71	14.79		82.18	60.56	29.57	
Trade/Some College	33.54	35.43	49.11		12.00	29.58	31.2	
Bach. Degree or above	59.92	56.86	36.09		5.82	9.86	39.23	
Income (%)								
<20K $	8.09	9.33	29.61		79.05	62.37	31.11	
20–59K $	35.06	46.94	48.68		18.58	31.18	35.97	
60K+ $	56.85	43.73	21.71		2.37	6.45	32.92	
Under lipid lowering treatment (%) (any time during trial period)	14.53	18.29	60.95		29.09	43.46	27.58	
Race (%)								
White	65.68	72.86	57.74		4.73	30.05	50.03	
Hispanic	10.74	10.86	22.02		89.09	45.54	31.6	
Other	23.58	16.29	20.24		6.18	24.41	18.37	
Smoking Status								
% Never smoker	63.29	64.29	49.11		65.82	49.53	60.39	
% Current smoker	3.38	4.00	0.00		11.27	11.21	5.74	
% Former smoker	33.33	31.71	50.89		22.91	39.25	33.87	
Packyrs, mean ±sd (range)	6.3±13.8	7.86±17.25		8.10±15.85	6.74±15.23	10.60±21.36		7.55±16.39
	(0–92)	(0–135)		(0–78)	(0–99)	(0–126)		(0–135)
Height (mean ±sd; range); in meters	1.70±0.10	1.69±0.10		1.60±0.07	1.60±0.10	1.55±0.07		1.65±0.11
	(1.36–1.94)	(1.42–1.98)		(1.40–1.80)	(1.37–1.92)	(1.27–1.70)		(1.27–1.98)
BMI (mean ±sd; range)	28.19±4.97	27.68±4.7		28.86±5.41	32.19±5.93	30.39±5.58		29.21±5.49
	(4.97–16.76)	(4.7–16.12)		(5.41–18.4)	(5.93–19.37)	(5.58–18)		(5.49–16.12)
LDL cholesterol (mean ±sd; range) mg/dL	128.79±30.38	147.43±26.41		162.85±26.39	99.59±29.46	146.23±38.69		134.37±36
	(56.4–315.6)	(61.2–277.8)		(90–256)	(25–198.8)	(56–280)		(25–315.6)
Cholesterol (mean ±sd; range) mg/dL	212.2±34.75	231.03±30.88		247.55±31.39	171.04±34.14	234.24±44.11		216.28±42.55
	(121–437)	(125–394)		(162–342)	(94–330)	(127–392)		(94–437)
Physical activity (in Met Cal) mean ±sd; (range)	2904.77±630.85	2854.9±725.57		2380.73±704.08	2821.18±727.67			2841.23±696.44
	(1466.9–5559.0)	(1196.9–6269.9)		(389–3641)	(1600.1–5988.0)			(389–6269.9)
Diastolic blood pressure, mean ±sd (range); mmHg	79.82±8.29	76.69±9.32		77.6±7.56	76.19±9.41	76.48±10.16		77.67±9.08
	(58–119.5)	(45–103)		(57–95)	(56–109)	(51–117)		(45–119.5)
Systolic blood pressure mean ±sd (range); mmHg	128.66±14.66	128.03±16.49		127.99±14.07	130.86±19.09	141.74±21.49		130.73±17.62
	(95.5–178.5)	(91–189)		(94–161)	(89–180)	(89–230)		(89–230)

**Figure 1:**
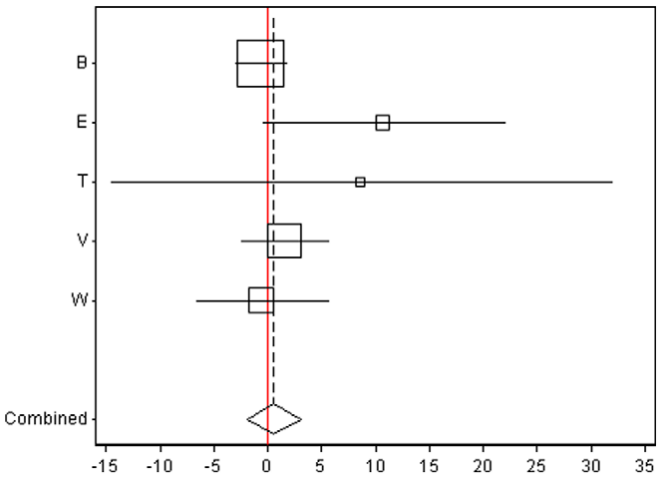
Association (with 95% CI) between a contrast in 10 ug/m^3^ home outdoor PM2.5 and CIMT progression by trial and the respective meta-analytic estimates. The X-axis indicates the regression coefficient (change) in micrometers. Studies: B  =  BVAIT, E  =  EPAT, T  =  TART, V  =  VEAPS, W  =  WELLHART. The solid line indicates the level of no effect.

**Figure 2:**
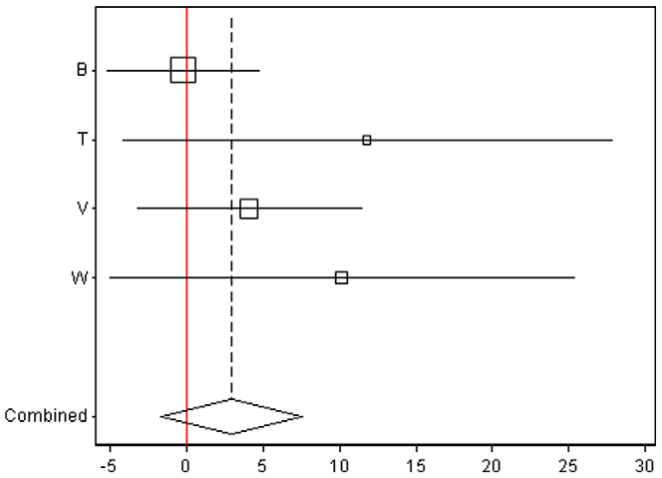
Associations (with 95% CI) of living within 100 m of a highway and CIMT progression by trial and the respective meta-analytic estimate. The X-axis indicates the regression coefficient (change) in micrometers. Studies: B = BVAIT, T = TART, V = VEAPS, W = WELLHART. Among the EPAT participants, no subject lived within 100 m of a major road. The solid line indicates the level of no effect.

The main results regarding air pollution and CIMT progression are presented in Table 3, Figure 1 and Figure 2. In general, both proximity markers of exposure and P2.5 were positively associated with CIMT progression. In the total sample, associations were statistically significant for proximity to highways and of borderline significance for both, PM2.5 (P-value  = 0.8) and living within 100 m of a highway *or* 50 m of a main road (p-value: 0.07; see Table 4). For those living within 100 m of a highway (N = 23), the CIMT progression was 5.62 µm/year (95% CI: 0.31–10.94; unadjusted model) and 5.46 µm/year (95%CI: 0.13–10.79; main model) faster than among those living further away (Figure 2). As expected, defining a less extremely polluted group as “exposed” (i.e. those living within 50 m of a main road *or* within 100 m of a highway; N = 238), the main model coefficient was lower (1.64; 95% CI: −0.15 to 3.42) (see Table 4). An increase of 10 µg/m3 in the home outdoor PM2.5 concentration was associated with a statistically insignificant 1.74 and 2.53 µm/year faster progression in CIMT in the unadjusted and the main adjusted model, respectively (Figure 1).

**Table 3:** Difference in CIMT progression (in micrometers per year) associated with a 10 ug/m^3^ contrast in PM2.5 and ‘living within 100 meters of a highway, respectively.

Covariates included in model	PM2.5			Within 100 m of traffic		
	Coeff	95% CI	p-value	Coeff	95% CI	p-value
**1-Pollutant models**						
No covariates (crude association)	1.74	−1.04, 4.51	0.219	5.62	0.31, 10.94	0.038
Baseline IMT	1.96	−0.82, 4.74	0.167	5.84	0.53, 11.16	0.031
On-trial treatment status	1.75	−1.02, 4.52	0.216	5.50	0.19, 10.81	0.042
On-trial treatment status, smoking, education	2.11	−0.72, 4.95	0.144	5.89	0.55, 11.23	0.031
Above + lipid lowering tx	2.43	−0.4, 5.26	0.092	5.63	0.3, 10.95	0.038
Treatment status, smoking, wine consumption, height, education, lipid lowering tx (MAIN MODEL)	2.53	−0.31, 5.38	0.081	5.46	0.13, 10.79	0.044
MAIN + baseline IMT	2.64	−0.20, 5.49	0.069	5.59	0.27, 10.92	0.040
MAIN + MET cal, menopause, diabetes, ETS work	1.91	−0.94, 4.77	0.189	5.40	0.1, 10.71	0.046
Above + HDL, high blood pressure	1.96	−0.9, 4.82	0.179	5.35	0.05, 10.66	0.048
MAIN + ‘trial’ as fixed effect	1.16	−1,71, 4.02	0.428	5.18	−0.10, 10.46	0.054
MAIN + ‘trial’ as random effect	1.29	−1.57, 4.15	0.377	5.21	−0.07, 10.49	0.053
**2-Pollutant models (PM2.5 and proximity to traffic):**						
MAIN MODEL	2.40	−0.44, 5.24	0.098	5.25	−0.08, 10.58	0.054
MAIN + ‘trial’ as fixed effect	1.30	−1.55, 4.15	0.371	5.00	−0.28, 10.29	0.064
MAIN + ‘trial’ as random effect	1.43	−1.42, 4.28	0.324	5.04	−0.24, 10.32	0.062

All models are based on the total population (N = 1483).

**Table 4:** Association between both markers of air pollution and CIMT progression among subgroups.

Subgroups	PM2.5				within 100 m traffic				within a highway (100 m)OR within a major road (50 m)			
	Coeff	95% CI	p-value	p-inter	Coeff	95% CI	p-value	p-inter	Coeff	95% CI	p-value	p-inter
**Total sample (same as ** **Table 3** **) (N = 1483)**	2.53	−0.31, 5.38	0.081	N/A	5.46	0.13, 10.79	0.044	N/A	1.64	−0.15, 3.42	0.072	N/A
**By Treatment group (trial treatment):**												
Placebo (N = 706)	0.88	−3.16, 4.92	0.670	0.457	0.96	−6.20. 8.12	0.793	0.046	1.88	−0.65, 4.41	0.146	0.707
Treatment (N = 777)	4.37	0.37, 8.37	0.032		11.43	3.46, 19.39	0.005		1.40	−1.12, 3.92	0.277	
**By income:**												
Less than 20K (N = 429)	4.55	−5.07, 14.16	0.354	0.446	13.61	2.17, 25.04	0.020	0.037	6.13	2.32, 9.94	0.002	<0.001
20K+ (N = 950)	1.91	−0.67, 4.49	0.146		1.21	−4.22, 6.65	0.662		−1.49	−3.38, 0.39	0.120	
**By education:**												
Some high school (N = 438)	3.02	−7.67, 13.7	0.579	0.614	12.23	1.47, 22.99	0.026	0.053	5.32	1.16, 9.47	0.012	0.001
Trade/some college & bachelor or graduate (N = 1043)	2.13	−0.38, 4.63	0.096		0.58	−5.23, 6.39	0.846		−0.57	−2.37, 1.22	0.530	

Coefficients are in micrometers, per 10 ug/m^3^ PM2.5; ‘living within 100 m of a highway’ (N = 1.55%); and ‘living within 100 m of a highway *or* within a major road (50 m)’ (N = 238), respectively. (main model as in Table 3). P-inter is the p-value under the null hypothesis that the main effects are the same in the two subgroups.

Main effect estimates were not appreciably altered by inclusion or exclusion of a range of covariates or adjustment for baseline CIMT, and adjusted coefficients were without exception slightly larger than the crude associations. Baseline CIMT and progression were mildly negatively correlated (r = −0.09; p<0.01). Adding both, PM2.5 and proximity to a highway to the main model had little impact on the coefficients although confidence intervals slightly increased (Table 3).

**Figure 3:**
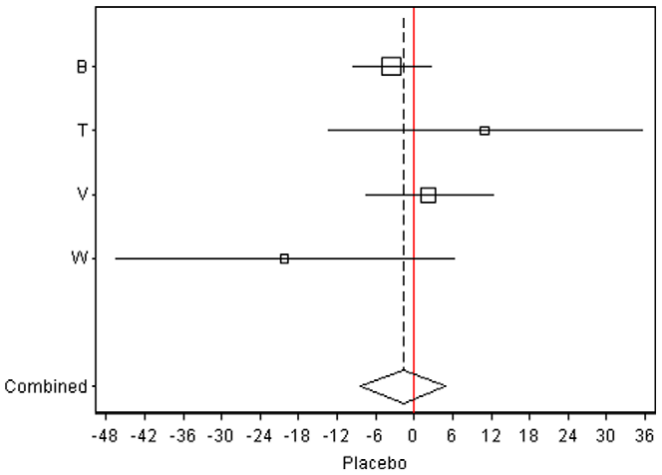
Associations (with 95% CI) of living within 100 m of a highway and CIMT progression and the respective meta-analytic estimates among the on-trial placebo group (treatment group: see Figure 4). EPAT: no subject lived within 100 m of a highway. The X-axis indicates the regression coefficient (change) in micrometers. The solid line indicates the level of no effect. Studies: B = BVAIT, T = TART, V = VEAPS, W = WELLHART.

**Figure 4:**
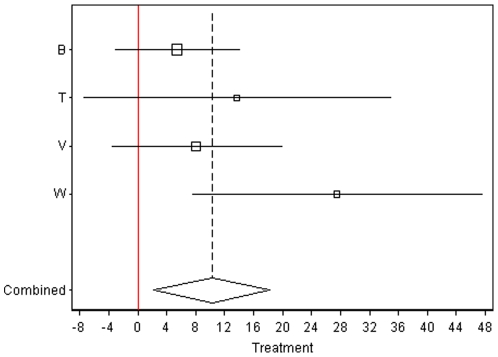
Same as Figure 3, but for the on-trail treatment group.

Table 4 (and Table S3 in the supplement) present the investigation of possible interactions between air pollution and other factors. The association of CIMT with ‘proximity to highways’ as well as with ‘proximity to highways (100 m) *or* a main road (50 m)’ was substantially larger and statistically significant among the socially deprived (as measured by low income). These effect modifications were statistically significant (p for interaction <0.05). A similar pattern emerged for PM2.5 but associations and interactions were not statistically significant (Table 4). Stratification by randomization status (active intervention vs. placebo) revealed larger and statistically significant coefficients for both, proximity to highways (100 m) and PM2.5, among the subjects randomized to active treatments. For the highway proximity, this interaction was statistically significant. The coefficients in the placebo group were not statistically significant and were five to ten times smaller relative to the treatment group (see Table 4 and Figure 3 & 4).

Associations did not differ by sex, lipid-lowering treatment, and ethnicity (Table S3 in supplement). Figures 1, 2, 3 and 4 show the main results by trial and the respective meta-analytic estimates. While air pollution coefficients appeared to be different across trials, power was low to formally test heterogeneity. To get an impression of the impact of pooling diverse trials we tested the main model in all possible combinations of pooling only four of the five trials. As shown in Table S3 (supplement), BVAIT and TART, in opposing directions, appeared to be the most influential trials. Namely, in an analysis based on only four trials, excluding BVAIT data, the size of the coefficients almost doubled, with significant adjusted coefficients for both, PM2.5 (5.27; 95% CI: 0.73, 9.80; p = 0.02) and highway proximity (9.14; 1.33 to 16.96; p = 0.02; Table S3); excluding instead TART data resulted in the smallest coefficients (Table S3).

## Discussion

This is the first study in humans to investigate the association between markers of exposure to ambient air pollution and the progression of CIMT, an accepted measure of the progression of atherosclerosis [26], [27]. CIMT results from the cumulative atherogenic processes that occur in the artery wall. As such, CIMT progression is associated with future clinical cardiovascular events [26], [28], [29]. Our data indicate that the progression of sub-clinical atherosclerosis correlates with home outdoor air quality, with particularly strong associations among those living along highways. Southern California highways have exceptionally high traffic density (i.e. several hundred-thousand vehicles per day) – several fold higher then on main surface roads – and most highways are designated truck routes. Moreover, trucks are the key source of diesel particles while passenger cars operate primarily with gasoline in North America. These features may explain the extreme gradients in ultrafine particles and other primary pollutants observed along Southern California highways with some ten-fold concentrations reported within the first 30 meters as compared to the background levels measured at >150–200 meters [18]. In line with the difference in traffic density – thus lower pollution - our finding of smaller effect estimates if the ‘exposed’ included 215 subjects living within 50 m of a main road is plausible.

While our progression findings have not been previously reported, the results agree with cross-sectional observations in humans [8], [9] and with several prospective animal experiments where rats, mice, and rabbits exposed to ambient particulate matter developed atherosclerotic plaques and calcification [5], [6], [7], [30]. These animal studies – one placing mice adjacent to a Southern California highway [6] – reported a range of effects relevant to atherogenesis and significant progression of atherosclerosis, which was possibly modified by endogenous or exogenous factors such as genetics and diet [7].

Despite pooling five trials, the major weakness of the study remains the limited sample size. The percent of people living very close to highways is small, in general, and was only 1.6% in our more affluent population. Thus, the strongest results relied on only 23 ‘exposed’ subjects (i.e. within 100 m of a highway). In contrast, results of the PM2.5 analyses were not affected by this limitation as this exposure metric is defined on a continuous scale. The PM2.5 estimates did not reach statistical significance in the full data, but were clearly significant among the pool of four instead of five studies (excluding BVAIT) as well as among the on-trial treatment group. In fact, the most intriguing and challenging aspect of our results are the sub-group findings. All subgroups tested were identified *a priori*, based on prior studies and hypotheses, and the confirmation of modifiers of effects underscores the plausible notion that there exist groups who are more susceptible to oxidative and inflammatory air pollutants. In line with other U.S.-based air pollution studies, the effects were stronger among the socioeconomically disadvantaged, a possible marker for concomitant adverse environmental exposures, poor diet, and a more stressful life [19].

In line with our previous cross-sectional findings, proximity to highways appeared to be more strongly related to CIMT progression in women but the difference did not reach statistical significance (Table S3 in the supplement). With the limited sample size, it was however difficult to evaluate this further. Similarly, to evaluate whether the apparent but not significant differences in the trial-specific estimates reflect differences in the distribution of susceptibilities among the populations, larger sample size would be needed. The trials had high levels of quality control and identical standards that yield a high degree of comparability in the CIMT data across all five trials [17], [26]. However, the different objectives and consequent recruitment procedures of each trial – all based on volunteers – increases the inherent heterogeneity in our data and limits our ability to generalize results.

The interaction between randomized ‘treatment on trial’ and the atherogenic effect of air pollution was apparently present across heterogeneous interventions ranging from vitamins (VEAPS and BVAIT) to the regulation of diabetic metabolism (TART) and hormones (EPAT and WELL-HART) (Figures 3 and 4). Given the double-blind randomized design applied in all trials, and treatment status indeed not being associated with pollution it is not very plausible to explain the interaction finding as chance alone.

With the exception of WELLHART, treatments affected CIMT progression in all trials although not necessarily in the entire populations [11], [12], [13], [14], [15]. This means that the treatments did affect vascular metabolism and homeostasis in a way that resulted in changes in the artery wall structure, measured with CIMT progression. Periods of metabolic change may be a vulnerable window in the presence of other pro-atherogenic factors [31], such as hypothesized for ambient particulate matter [2], [4], [32]. All interventions used in these trials have both pro- and anti-atherogenic properties, and the postulated oxidative and inflammatory effects of ambient air pollutants may interact with the metabolic effects of these interventions in a complex manner. Congruent with this hypothesis are studies in mice where effects of ambient particulate matter on atherogenesis were stronger among those fed with a high fat chow as compared to those under a normal diet [7]. One would need larger studies to corroborate these interactions for the various on-trial treatments. Moreover, the interaction by treatment status was only apparent for PM2.5 and ‘living close to highways’ (with 23 subjects ‘exposed’), but not so for ‘living close to highways or main roads’. In light of this inconsistency, one may question a causal interpretation of the observed effect modification due to treatments. However, as mentioned above in the Methods, living within 50 m of main roads includes 215 subjects in the ‘exposed’ group with lower traffic-related pollution than those 23 participants living within 100 m of a highway. Thus, effect stimates would be expected to be smaller. Statistical power to evaluate interactions for this marker of exposure is not sufficient.

Similarly, due to limited sample sizes, the statistically significant interaction between on-trial treatment and the effects of traffic-related pollution cannot be evaluated further in each trial separately. Thus, it is not clear whether the same interactions would prevail in larger studies or be restricted to a few on-trial treatments. Below, we conjecture about explanations of this interaction trial by trial.

Air pollution associations were strongest in TART with only slightly larger associations in the treatment group. All TART subjects were patients with type 2 diabetes – a pathology with a well known atherogenic propensity and with some preliminary evidence of higher susceptibility to adverse cardiovascular effects of air pollution [4], [11]. The trial tested a peroxisome proliferator-activated receptor agonist (PPARγ), namely thiazolidinedione (TZD). Activation of PPARγ inhibits inflammatory cytokines, cellular proliferation and migration, and expression of cellular adhesion molecules. However, the stimulation of macrophages into foam cells by promoting lipid uptake is a known pro-atherogenic property of PPARγ activation. These effects may dominate if exposure to air pollution is increased, e.g., recruitment of precursors of macrophages as observed in rabbits [5] or the oxidation of lipids [6], [33]. As shown in TART, the balance between pro- and anti-atherogenic effects of TZD depends on host factors and this may hold true for the interaction with air pollution as well. In fact, TZD had protective effects only among those with advanced atherosclerosis (i.e. thicker CIMT) at baseline, and the considerable heterogeneity of responses to TZD supports the notion of complex interactions between exogenous factors (e.g. TZD or air pollution) and reactions in the vascular bed [34].

VEAPS tested the atherogenic effects of vitamin E supplementation. While known as an anti-oxidant, vitamin E also has pro-oxidant and pro-atherogenic effects, and those appeared to dominate in the treatment group where CIMT progression was 4.0 versus only 2.3 micrometers per year in the placebo group (p = 0.08) [15]. Oxidized vitamin E may act as a pro-oxidant in the artery wall and these proatherogenic properties may be amplified by pro-inflammatory effects of air pollution. Similarly, interactions between treatment (vitamin B), other endogenous factors and environmental effects may apply to BVAIT; effects of vitamin B were limited to individuals with elevated baseline homocysteine levels. Homocysteine is linked to atherogenic pathways, and interactions between ambient air pollution and homocysteine have been reported previously [35].

EPAT tested the effects of unopposed estrogen replacement therapy (ERT) with 17-beta-estradiol among postmenopausal women [12]. ERT significantly reduced CIMT progression by 5 micrometers per year, but this beneficial effect was seen only in women who did not receive lipid-lowering treatment, namely with statins whereas among this latter group, CIMT changed far less during the EPAT follow-up. Whether the somewhat larger PM2.5 coefficient in the treatment group was a chance finding, and how it interacted with lipid-lowering treatment cannot be determined with the available sample size. However, it is well known that the atherogenic effects of hormones underlie rather complex mechanisms which depend on a range of host factors including time since menopause, concomitant diseases, or type, dose and route of drug administration [36].

The WELLHART trial tested hormones in women with established coronary artery disease, at a later stage of menopause [13]. While treatment had no effect on coronary artery atherosclerosis, the interaction of air pollution with on-trial treatment status was also substantial in this population. The interaction between hormonal factors, ambient air pollutants, and atherogenesis needs further investigation.

An interesting finding is the similarity of coefficients from the one-pollutant and two-pollutant models. As mentioned in Methods, the derivation of the PM2.5 surface did not include any markers of local traffic while the proximity measures capture this local exposure space. Thus, the two-pollutant model results suggest independent associations of ambient PM2.5 (albeit not statistically significant in all groups) and proximity to traffic with atherosclerosis (Table 3). This is in line with two different interpretations. First, it supports the idea that different components of the complex mixture contained in urban air pollution act, in part, independently through complementary mechanisms [4], [32]. Proximity to highways may be a marker for exposure to high loads of ultrafine particles and other highly redox-active pollutants, in particular diesel particles [37], and constituents affecting the airways and alveoli leading to systemic inflammatory responses [38] and atherogenesis [6]. The larger particles (i.e. PM2.5) may result in inflammatory reactions in the small and upper airways alike, and both types of pollution may independently enhance systemic inflammation [38]. Second, the independent effects may indicate that both exposure assessment approaches capture similar types of pollution but on different spatial scales and concentration levels; ‘proximity’ would characterize the most extreme local conditions (hot spots) while PM2.5 captures the additional contrast occurring between geographic areas [37]. In contrast to other areas, e.g. the U.S. East coast, PM2.5 in the Los Angeles area, is to a large extent the consequence of primary and secondary pollutants from traffic, and the proximity measure is, by definition, a marker for traffic-related exhaust emissions and/or re-suspended local pollutants. The results, therefore, point in the direction of traffic-related pollution being an atherogenic health hazard. This is in line with the Southern Californian animal studies where experimental chambers were placed adjacent to a highway. The traffic-related particles enhanced atherogenesis in mice [6].

Longitudinal analyses of change in biologic markers raise some contentious statistical issues with regard to the adjustment for the CIMT levels observed at baseline [39], [40]. Our model estimates were not appreciably altered by adjustment for baseline CIMT and results were very stable across a range of modelling assumptions; thus, the findings are unlikely the result of modelling artefacts. In general, we used baseline covariate information to adjust the multivariate models rather than the change in some of these variables. However, individual follow-up time was fairly short (2–3 years); thus, change in lifestyles or other factors are not expected to be substantial. We have some indirect evidence that a potential change in an important lifestyle, namely giving up smoking during follow-up, was probably not relevant in our analyses; associations of pollution with CIMT progression was not different in smokers, and only 6% of participants were smokers at baseline.

Our analyses do also not take into account possible changes in exposure due to change in pollution levels over time. Exposure was assigned based on the year 2000 information. While levels of pollution fluctuate year by year, with general downward trends, over decades, the key question relevant for our analyses is whether the spatial contrasts of the year 2000 would reflect those of other years. Fortunately, spatial contrasts are more stable over time and many long-term air pollution studies capitalize on the fact that spatial contrasts and traffic data of a specific year will properly approximate exposure contrasts for several years. We are unable to investigate the possible impact of this type of exposure misclassification because departure of assessment from the year 2000 is, in our case, a correlate of *trial* as each trial took place in different periods (shown in Table 1). As discussed, trials are inherently different in many other respects.

We had no noise data available, thus, a full assessment of the correlations between noise and pollutants – in some studies reported to be lower than expected [41] – was not possible, nor could we evaluate the potential confounding of traffic-relate noise. Future studies should investigate this in more detail.

We were also not able to integrate in the exposure assignment changes in residence that may have occurred during the trial follow-up. This information was not available nor could we approach all former participants as some trials happened several years ago. However, six years of residential history was available among a subsample of 245 subjects. The vast majority (N = 210) had not moved for six years; we therefore conclude that exposure misclassification due to ignored moving patterns were not a major limitation. Moreover, it is unlikely that moving patterns were jointly correlated with CIMT progression and air pollution at the address used in our exposure assignment, thus, biases from this exposure misclassification would be expected to go toward null.

CIMT is the earliest detectable anatomic change in the development of atherosclerosis and progression of CIMT is predictive of clinical cardiovascular events [28], [29]. The magnitude of our effect estimates was similar to the effects of on-trial treatments on CIMT progression. Comparison is, however, hampered by the differences in absolute progression between trials due to the differences in inclusion criteria. The mean effect of a 5.4 micrometer greater progression per year among those living within 100 m of busy roads or per 23 ug/m^3^ contrast in PM2.5 is approximately twice the average progression as observed across these trials. Our results for a 10 ug/m^3^ contrast in PM2.5 being associated with 2.5micrometer (total data) to 4.4micrometer (treatment groups) increase in annual CIMT progression would translate into 10–30% higher rates in coronary events [26]. This is well within the range of the results reported by the Women's Health Initiative where cardiovascular events increased by 24% (95%CI: 9–41%) for the same contrast of 10 ug/m^3^ ambient PM2.5 [42]. While these studies and settings are different, our observed effect appears to be of biologically plausible size [13].

Interactions between anti-atherogenic treatments such as lipid-lowering prescriptions and ambient air pollution may be a major concern and research challenge as some treatments may influence mechanisms relevant in the effects of air pollution. As we used existing data we were unable to fully investigate whether such prescriptions taken at baseline or during follow-up or both interacted with the main effects. As shown in the supplement (Table S3), subjects reporting lipid-lowering treatments at study entry showed substantially stronger associations between CIMT progression and pollution than those without such treatments or those who newly received lipid lowering prescriptions during the follow-up period of the trial. Again, statistical power was insufficient to further elaborate on these patterns which may also be a chance finding.

In sum, this study indicates an association between exposure to traffic-related air pollution and the progression of atherosclerosis in humans. While in agreement with cross-sectional findings and animal studies, these results need to be corroborated with longitudinal studies particularly designed to evaluate this hypothesis, and to investigate the role of endogenous and exogenous co-factors that may interact with the vascular toxicity of ambient pollutants. Future studies should investigate whether atherogenic effects of air pollution may be larger in women, among the socially deprived or poorly educated, and possibly among those under treatments that interact with atherogenic pathways. The issue is of substantial public health relevance due to the high burden of morbidity and mortality related to atherosclerosis, and the very high number of people exposed to ambient air pollutants through the entire lifecourse.

## Supporting Information

Text S1 Supporting material text.
(0.04 MB DOC)

Table S1 Missing variable information subject to imputation.
(0.06 MB DOC)

Table S2 Description of the exclusion criteria of B-Vitamin Atherosclerosis Intervention Trial (BVAIT), the Vitamin E Atherosclerosis Prevention Study (VEAPS), the Estrogen in the Prevention of Atherosclerosis Trial (EPAT), the Troglitazone Atherosclerosis Regression Trial (TART), and the Women's Estrogen-Progestin Lipid-Lowering Hormone Atherosclerosis Regression Trial (WELL-HART) used in this analyses. (CVD  =  cardiovascular diseases; LDL  =  low density lipids). For inclusion criteria and main references: see Table 1 in the main manuscript.
(0.05 MB DOC)

Table S3 Association between the main markers of air pollution and CIMT progression among subgroups defined by sex, reported lipid-lowering medication, and after exclusion of one or two trials, with p-values for interaction. Coefficients are in micrometers, per 10 ug/m3 PM2.5 or ‘living within 100 m of a highway’, respectively. (Main model same as in Table 3).
(0.07 MB DOC)
