# Correction: Ambient Air Pollution and the Progression of Atherosclerosis in Adults

**DOI:** 10.1371/annotation/21f6b02b-e533-46ca-9356-86a0eef8434e

**Published:** 2010-03-03

**Authors:** Nino Künzli, Michael Jerrett, Raquel Garcia-Esteban, Xavier Basagaña, Bernardo Beckermann, Frank Gilliland, Merce Medina, John Peters, Howard N. Hodis, Wendy J. Mack

An affiliation was omitted. The additional affiliation, which is for first author Nino Künzli, is University of Basel, Basel, Switzerland.

