# Supplementary material for: Ambient Air Pollution and the Progression of Atherosclerosis in Adults
Source: PLoS One. 2010 Feb 8;5(2):e9096. doi: 10.1371/journal.pone.0009096 (PMC2817007; doi:10.1371/journal.pone.0009096)
Supplement: Text S1 — Supporting material text. (0.04 MB DOC) [file pone.0009096.s001.doc]

**ONLINE SUPPLEMENT**

**Ambient Air Pollution and the Progression of Atherosclerosis in Adults**

Nino Künzli ^1,2)^, Michael Jerrett ^3)^, Raquel Garcia ^2)^, Xavier Basagaña ^2)^, Bernardo Beckermann ^3)^, Frank Gilliland ^4)^, Merce Medina ^2)^, John Peters ^4)^ Howard N. Hodis ^4,5)^, Wendy J. Mack ^4)^

1) Swiss Tropical and Public Health Institute Basel, Switzerland

2) Centre for Research in Environmental Epidemiology CREAL Barcelona, Spain

3) Division of Environmental Health Sciences, School of Public Health, University of California, Berkeley, USA

4) Department of Preventive Medicine University of Southern California Los Angeles, USA

5) Atherosclerosis Research Unit, Department of Medicine University of Southern California Los Angeles

**Imputation of missing values**

The following variables had no missing covariate data in any of the subjects: age, sex, BMI, height, lipid lowering treatment (yes/no; ever/current), anti-inflammatory treatment; randomization status, asthma, wine consumption, and living. Covariates with missing data are shown in Table S1. The most common missing relate to passive smoke exposure at work, and level of physical activity, which were not available in all trials. Income was missing in 104 subjects but educational data was instead available for all but 2. The main model shown in the main paper included only 5 subjects with some imputed covariates; thus, results among those with complete data and the total sample including the five were almost identical.

Imputation was done in the following way: The *ice* Stata command (Multiple Imputation by Chained Equation) was used. Imputed values on *menopaus* and *numwine* were conditional on sex and wine respectively. Because former smokers could have *packyrs* equals 0, former and current smokers sample was used for imputing values to ln(packyrs+1). Five imputations were obtained, and *micombine* command was used to obtain combined estimates (and their standard errors) across replicates obtained previously by multiple imputation.
