# Supplementary material for: Ambient Air Pollution and the Progression of Atherosclerosis in Adults
Source: PLoS One. 2010 Feb 8;5(2):e9096. doi: 10.1371/journal.pone.0009096 (PMC2817007; doi:10.1371/journal.pone.0009096)
Supplement: Table S1 — Missing variable information subject to imputation. (0.06 MB DOC) [file pone.0009096.s002.doc]

Table S1
