# Supplementary material for: Ambient Air Pollution and the Progression of Atherosclerosis in Adults
Source: PLoS One. 2010 Feb 8;5(2):e9096. doi: 10.1371/journal.pone.0009096 (PMC2817007; doi:10.1371/journal.pone.0009096)
Supplement: Table S2 — Description of the exclusion criteria of B-Vitamin Atherosclerosis Intervention Trial (BVAIT), the Vitamin E Atherosclerosis Prevention Study (VEAPS), the Estrogen in the Prevention of Atherosclerosis Trial (EPAT), the Troglitazone Atherosclerosis Regression Trial (TART), and the Women's Estrogen-Progestin Lipid-Lowering Hormone Atherosclerosis Regression Trial (WELL-HART) used in this analyses. (CVD = cardiovascular diseases; LDL = low density lipids). For inclusion criteria and main references: see Table 1 in the main manuscript. (0.05 MB DOC) [file pone.0009096.s003.doc]

| Characteristic | BVAIT | VEAPS | EPAT | TART | WELLHART |
| --- | --- | --- | --- | --- | --- |
| Main exclusion Criteria: Diseases | Diabetes, CVD, cancer, untreated Thyroid disease | Diabetes, CVD, Thyroid disease | thyroid disease, breast cancer, CVD | Life-threatening diseases | Thyroid disease |
| Blood pressure | Syst > 160 and /or diast>100 mmHg | Diastolic >100mmHg | Diastolic>110 mmHg | Syst >=170mmHg or diast>=110mmHg | Diastolic >110 mmHg |
| Serum creatinine | >1.6 mg/dL | >0.74 mg/Dl | >2.5mg/dL | >2.0mg/dL | >2.5mg/dL |
| Triglyceride level | > 150 mg/dL | N/A | N/A | >=1000mg/dL (fasting) | N/A |
| Fasting glucose | > 140 mg/dL | N/A | >200mg/dl | N/A | >150 mg/dL |
| Alcohol / smoking | High alcohol intake (>5 drinks/day) | High alcohol intake (>5 drinks/day) | current smoker | High alcohol intake (>5 drinks/day) | >15 cigarettes/day |
