# Supplementary material for: Ambient Air Pollution and the Progression of Atherosclerosis in Adults
Source: PLoS One. 2010 Feb 8;5(2):e9096. doi: 10.1371/journal.pone.0009096 (PMC2817007; doi:10.1371/journal.pone.0009096)
Supplement: Table S3 — Association between the main markers of air pollution and CIMT progression among subgroups defined by sex, reported lipid-lowering medication, and after exclusion of one or two trials, with p-values for interaction. Coefficients are in micrometers, per 10 ug/m3 PM2.5 or ‘living within 100 m of a highway’, respectively. (Main model same as in Table 3). (0.07 MB DOC) [file pone.0009096.s004.doc]

| Subgroups | PM2.5 | | | | Within 100m traffic | | | |
| --- | --- | --- | --- | --- | --- | --- | --- | --- |
|  | Coeff | 95% CI | p-value | p-inter | Coeff | 95% CI | p-value | p-inter |
| **By sex** |  |  |  |  |  |  |  |  |
| Men (N=546) | 1.72 | -2.08, 5.51 | 0.375 | 0.704 | 1.65 | -5.35, 8.65 | 0.644 | 0.186 |
| Women (N=937) | 3.05 | -0.96, 7.06 | 0.136 |  | 8.72 | 1.14, 16.31 | 0.024 |  |
| **By lipid lowering medication: #)** |  |  |  |  |  |  |  |  |
| Never (N=1074) | 1.01 | -2.01, 4.03 | 0.513 | 0.080 | 4.56 | -1.60, 10.71 | 0.147 | 0.720 |
| Past (N=286) | 10.85 | 0.94, 20.77 | 0.032 |  | 10.65 | -7.72, 29.02 | 0.255 |  |
| During trial (N=123) | 2.50 | -6.69, 11.68 | 0.591 |  | 6.93 | -4.59, 18.44 | 0.236 |  |
| **By groups of trials** |  |  |  | P *) |  |  |  | P *) |
| 4 trials (excl. BVAIT) (N=1008) | 5.27 | 0.73, 9.80 | 0.023 | 0.599 | 9.14 | 1.33, 16.96 | 0.022 | 0.656 |
| 4 trials (excl. TART) (N=1208) | 1.38 | -0.87, 3.64 | 0.227 | 0.104 | 2.77 | -2.13, 7.68 | 0.267 | 0.232 |
| EPAT, TART & WELLHART (N=658) | 7.29 | 0.39, 14.19 | 0.038 | 0.564 | 11.52 | 0.15, 22.90 | 0.047 | 0.928 |
| **By ethnicity:** |  |  |  |  |  |  |  |  |
| White (N=471) | 1.84 | -0.87, 4.55 | 0.184 | 0.733 | 1.30 | -6.59, 9.19 | 0.747 | 0.571 |
| Hispanic (N=468) | 3.72 | -5.55, 12.99 | 0.431 |  | 7.89 | -3.51, 19.3 | 0.174 |  |
| Other (N=272) | 4.55 | -2.4, 11.51 | 0.198 |  | 4.83 | -3.7, 13.35 | 0.266 |  |

***)** p-value for heterogeneity of main effects between those trials included in the respective models

#) lipid lowering medication: “past”: subjects reporting treatment at baseline, but not during the trial;

“during trial”: reporting treatment at one or more visit during baseline.
